# Supplementary material for: WordCommentsAnalyzer: A windows software tool for qualitative research
Source: F1000Res. 2018 Sep 4;7:536. Originally published 2018 May 3. [Version 2] doi: 10.12688/f1000research.14819.2 (PMC6137411; doi:10.12688/f1000research.14819.2)
Supplement: Supplementary file 3 [file f1000research-7-17614-s0002.tgz › b2a79496-4ada-47c4-9682-a99ce867836d.zip!/Eight Iranian Health Journals/Salmand_Iranian Journal of Ageing _5.docx]

© Salmand_Iranian Journal of Ageing . This work is licensed under CC BY-NC 4.0 (https://creativecommons.org/licenses/by-nc/4.0/).

Volume & Issue: دوره 12 - شماره 1

Title: بررسی مقایسه‌ای سطح ناتوانی در زنان و مردان سالمند تحت پوشش سازمان بازنشستگی شهرداری تهران در سال 1394

http://salmandj.uswr.ac.ir/article-1-1196-fa.html

اهداف با توجه به افزایش جمعیت سالمندان در کشور، این مطالعه با هدف تعیین میزان ناتوانی سالمندان صورت گرفت و ارتباط آن با جنسیت انجام شد. مواد و روش ها این پژوهش به روش توصیفی‌تحلیلی از نوع همبستگی بر روی ۳۰۰ نفر از سالمندان ۶۰ ساله و بالاتر تحت پوشش سازمان بازنشستگی شهرداری تهران به روش نمونه‌گیری تصادفی و از طریق مراجعه به در منازل افراد سالمند و مصاحبه با آنان صورت گرفته است. ابزار جمع‌آوری اطلاعات، پرسش‌نامه‌ای مشتمل بر دو بخش بود که بخش اول درباره مشخصات جمعیت‌شناختی، اقتصادی، اجتماعی و ابتلا به بیماری‌ها و بخش دوم، پرسش‌نامه WHODAS۲ از سازمان بهداشت جهانی بوده است. داده‌ها با استفاده از نرم‌افزار   SPSS۱۸ بررسی شدند. یافته ها میانگین سنی کل افراد مورد مطالعه ۷/۱۱±۶۸/۱۴ سال و میانه سن آنان ۶۶ سال بود. در میان سالمندان تحت مطالعه، ۱۷۵ نفر (۴۶ درصد) بدون ناتوانی بودند و ۱۹/۱۳ درصد افراد ناتوان (۱۲۵ نفر)، ناتوانی خفیف داشتند. بیشترین میزان ناتوانی در حیطه حضور در جامعه با میانگین ناتوانی (۵/۶) در زن‌ها و (۴/۳۸) در مردها دیده شد و حداقل میانگین ناتوانی در مردها (۰/۷۲) مربوط به حیطه مراقبت از خود و بهداشت شخصی و در زن‌ها (۱/۰۷) بوده است. نتیجه گیری سالمندان مطالعه حاضر در مقایسه با سایر کشورها ناتوانی کمتری داشتند، با این حال توجه به روند افزایش جمعیت سالمندان و سلامت و کاهش ناتوانی‌های آن‌ها ضروری به نظر می‌رسد. طبق بررسی انجام‌شده درصد ناتوانی در زنان (۵۸/۱۴ درصد) نسبت به مردها (۵۳/۳۱ درصد) بیشتر بود و ناتوانی با سن رابطه معنی‌داری داشت. (P<۰/۰۰۱)

Title: تأثیر هشت هفته برنامه تمرینی تحمل وزن اندام تحتانی و مصرف زیاد کافئین بر زمان واکنش زنان یائسه

http://salmandj.uswr.ac.ir/article-1-1219-fa.html

اهداف هدف از انجام این مطالعه تأثیر هشت هفته برنامه تمرینی تحمل وزن اندام تحتانی و مصرف زیاد کافئین بر زمان واکنش زنان سالمند بود.  مواد و روش ها این تحقیق از نوع نیمه‌تجربی بود که با استفاده از طرح تحقیق پیش‌آزمون و پس‌آزمون اجرا شد. شرکت‌کنندگان این پژوهش شامل ۴۳ زن یائسه سالمند غیرفعال با میانگین سنی ۸۸/۵±۶۹/۵۵ سال بودند که به صورت تصادفی به سه گروه فعال (۱۵نفر)، کافئین (۱۵نفر) و کنترل (۱۳نفر) تقسیم شدند. گروه فعال هشت هفته تمرین تحمل وزن را اجرا کردند، درحالی‌که گروه کافئین و کنترل هیچ گونه برنامه تمرینی یا تغذیه‌ای نداشتند. برای اندازه‌گیری شاخص‌های ترکیب بدن و زمان واکنش ساده و انتخابی آزمودنی‌ها در مراحل پیش‌آزمون و پس‌آزمون از دستگاه تحلیل‌گر بدن و دستگاه واکنش‌سنج استفاده شد. به منظور تحلیل آماری از آزمون‌های تی همبسته، تحلیل واریانس یک‌طرفه و آزمون تعقیبی توکی استفاده شد.  یافته ها نتایج نشان داد تمرین‌های تحمل وزن در مورد زمان واکنش ساده (نور) و زمان واکنش انتخابی معنادار بود، اما در مورد زمان واکنش ساده (صدا) معنادار نبود (به‌ترتیب P=۰/۰۰۳، P=۰/۰۰۳ ، P= ۰/۰۹). همچنین مشخص شد مصرف یک بار کافئین با غلظت ۲ میلی‌گرم به ازای هر کیلوگرم وزن بدن آزمودنی‌ها بر زمان واکنش آن‌ها تأثیری نداشت (۰/۱۲=P).  نتیجه گیری نتایج این تحقیق نشان داد فعالیت بدنی تحمل وزن از طریق تغییرات مثبت فیزیولوژیکی، زمان واکنش سالمندان را بهبود می‌بخشد

Title: بررسی و مقایسه روند سال‌خوردگی جمعیت در کشورهای آسیایی و اروپایی طی سال های 1950 تا 2015

http://salmandj.uswr.ac.ir/article-1-1200-fa.html

اهداف هدف از انجام این مطالعه بررسی و مقایسه روند سال‌خوردگی جمعیت در کشورهای اروپایی و آسیایی طی سال‌های ۱۹۵۰ تا ۲۰۱۵ است.  مواد و روش‌ها روش انجام این مطالعه به صورت تحلیل ثانویه است. داده‌های استفاده‌شده از داده‌های بخش جمعیت سازمان ملل متحد برای ۹۰ کشور اروپایی و آسیایی گرفته شده است. روش آماری استفاده‌شده، تحلیل پیشینه بقاست. داده‌ها با استفاده از روش آماری stata و روش‌های ناپارامتری برآورد تابع بقای کپلان میر، نلسن الن و مدل نیمه‌پارامتری مخاطره متناسب کاکس تحلیل شدند. یافته‌ها یافته‌ها نشان داد نرخ باروری کلی و امید زندگی در دو قاره اروپا و آسیا به سمت همگرایی پیش می‌رود. احتمال سال‌خوردگی با نرخ باروری کلی رابطه منفی و با امید زندگی رابطه مثبت دارد. برآورد تابع بقا و مخاطره تجمعی برای دو منطقه آسیا و اروپا در سال ۲۰۱۵ نشانگر آن است که احتمال سال‌خوردگی در این دو منطقه به طور معناداری متفاوت است. تابع بقای سال‌خوردگی برای کشورهای اروپایی به صفر نزدیک شده است؛ این بدان معناست که بیشتر کشورهای اروپایی وارد مرحله سال‌خوردگی جمعیت شده‌اند، درحالی‌که احتمال بقای سال‌خوردگی برای کشورهای آسیایی بیشتر از ۰/۷۵ است.  نتیجه‌گیری بر اساس نتایج این پژوهش، طی سال‌های ۱۹۵۰ تا ۲۰۱۵ درصد سال‌خوردگی جمعیت در همه مناطق قاره اروپا با فاصله فاحشی بیشتر از کشورهای آسیایی است. با توجه به کاهش سریع نرخ باروری و افزایش امید زندگی طی دهه‌های اخیر در مناطق کمتر توسعه‌یافته قاره آسیا، پیش‌بینی می‌شود روند سال‌خوردگی جمعیت بر خلاف دهه‌های گذشته در این مناطق سرعت بیشتری به خود بگیرد و درنهایت تا سال ۲۰۵۰، این شاخص در کشورهای آسیایی از کشورهای اروپایی پیشی بگیرد.

Title: تمرین‌پذیری عضلات تثبیت‌کننده کمری زنان سالمند در مقایسه با زنان جوان به دنبال تمرین مقاومتی و مصرف پروتئین سویا

http://salmandj.uswr.ac.ir/article-1-1245-fa.html

اهداف: تغییرات توده عضله اسکلتی یکی از مشکلات اجتناب‌ناپذیر سالمندی است و فرایندی چندبُعدی محسوب می‌شود که با کاهش بارز عملکرد عصبی‌عضلانی و جسمانی و ناتوانی افراد سالمند نسبت به افراد جوان همراه است. هدف مطالعه حاضر، تمرین‌پذیری عضلات تثبیت‌کننده کمری زنان سالمند در مقایسه با زنان جوان به دنبال تمرین مقاومتی و مصرف پروتئین سویا است.  مواد و روش ها: این مطالعه به صورت نیمه‌تجربی انجام شد. جامعه آماری شامل ۲۷ زن سالمند (۶۰/۱۶±۶۹/۳ سال) و ۱۰ زن جوان (۴/۱±۳۰/۰۸ سال) بود. افراد سالمند به طور تصادفی به گروه تمرین‌های پایدارکننده کمر با شیرسویا، گروه تمرین‌های پایدارکننده کمر و گروه کنترل (۹ فرد در هر گروه) تقسیم شدند و به مدت ۱۰ هفته و هر هفته ۳ جلسه، در مداخله ورزشی و تغذیه‌ای شرکت کردند. شرکت‌کنندگان شیرسویا را در سه وعده با فعالیت ورزشی و بدون فعالیت نوشیدند. مشخصات اولتراسونیک عضله مولتیفیدوس، میزان توانمندی (ODI)، عملکرد کمر (BPS) و وضعیت تغذیه زنان سالمند در دو مرحله قبل و بعد اندازه‌گیری و با تحلیل واریانس دوطرفه تجزیه‌وتحلیل شد.  یافته ها: سطح مقطع عضله مولتیفیدوس زنان سالمند (۰/۹۰۸±۳/۴۹ سانتی‌مترمربع) پیش از مداخله تفاوت معناداری با زنان جوان (۴۲/۵۲±۷/۱ سانتی‌مترمربع) داشت. در پایان مداخله در هر دو گروه تمرین‌های ثبات مرکزی با شیرسویا و تمرین‌های ثبات مرکزی نسبت به زنان جوان (به ترتیب ۱/۱۸±۵/۲۸ و ۰/۴۴±۵/۴۱ سانتی‌مترمربع) بهبود معناداری دیده شد (۰/۰۵>P)، اما این اختلاف حذف نشد. میزان توانمندی عملکرد کمر در دو گروه تمرین‌های ثبات مرکزی با شیرسویا و تمرین‌های ثبات مرکزی نسبت به گروه کنترل (به ترتیب ۲/۸±۱۱/۷۲- و ۳/۳±۱۰/۲- امتیاز) بهبود معناداری یافت (۰/۰۵>P).  نتیجه گیری: انجام ده هفته مداخله تمرین‌های پایدارکننده کمر و مصرف شیرسویا باعث افزایش مشخصات اولتراسونیک عضله مولتیفیدوس کمری زنان سالمند می‌شود و اختلاف سنی ایجادشده در سطح مقطع عضلانی را کاهش می‌دهد. از طرفی بهبود وضعیت عضله مولتیفیدوس نه‌تنها با بهبود توانمندی و عملکرد کمر همراه است، بلکه موجب بهبود استقلال فردی و مشارکت اجتماعی زنان سالمند نیز می‌شود.

Title: تأثیر هشت هفته تمرین مقاومتی بر هایپرتروفی و قدرت و مایوستاتین مردان سالمند و جوان

http://salmandj.uswr.ac.ir/article-1-1164-fa.html

اهداف با افزایش سن توده و قدرت عضلانی کاهش می‌یابد که به آن سارکوپنیا گفته می‌شود. تمرین مقاومتی یکی از روش‌های مؤثر برای مقابله با کاهش توده و قدرت عضلانی است. هدف این پژوهش بررسی تأثیر تمرین مقاومتی بر توده عضلانی و قدرت و غلظت مایوستاتین است. مواد و روش ها پس از فراخوان در سطح شهر اهواز، از بین افراد داوطلب برای شرکت در پژوهش، ۱۴ مرد سالمند (دامنه سنی ۵۵ تا ۷۰) و ۱۱ مرد جوان (دامنه سنی ۲۰ تا ۳۵) به صورت تصادفی انتخاب شدند. شرکت‌کنندگان در برنامه تمرین مقاومتی پیش‌رونده (افزایش تدریجی بار تمرین از هفته اول ۵۰ درصد یک تکرار بیشینه تا هفته هشتم ۸۵ درصد یک تکرار بیشینه)، سه روز در هفته و به مدت هشت هفته شرکت کردند. مقدار کالری دریافتی، قدرت، توده عضلانی، سطح مقطع عضلانی (با سی‌تی‌اسکن) و غلظت مایوستاتین قبل و بعد از دوره تمرین اندازه‌گیری شد. یافته ها نتایج نشان داد پس از هشت هفته، قدرت در گروه سالمند نسبت به گروه جوان افزایش بیشتری داشته است (P<۰/۰۵)، درحالی‌که توده عضلانی کل بدن فقط در گروه جوان افزایش یافت (P<۰/۰۵). میزان کالری برنامه غذایی دو گروه تفاوت معناداری نداشت (P>۰/۰۵). همچنین سطح مقطع عضله چهارسر ران در گروه جوان (۱۲/۲۱±۷۶/۰۳ سانتی‌متر مربع در پیش‌آزمون تا ۱۱/۵۴±۷۹/۱۶ سانتی‌متر مربع در پس‌آزمون) نسبت به گروه سالمند (۰۳/۷۵±۶۰/۱۰ سانتی‌متر مربع در پیش‌آزمون تا ۱۰/۴۲±۶۱/۹۳ سانتی‌متر مربع در پس‌آزمون) افزایش معناداری داشت (P<۰/۰۵). غلظت مایوستاتین (به ترتیب ۱/۸۹±۹/۸۶ و ۱/۶۶±۳/۶۸ نانوگرم بر میلی‌لیتر در گروه سالمند و جوان) پس از هشت هفته تمرین کاهش معناداری در دو گروه داشت (P<۰/۰۵)، اما بین دو گروه اختلافی مشاهده نشد (P>۰/۰۵). همچنین مایوستاتین با توده عضلانی سالمندان ارتباط معناداری داشت (P<۰/۰۵، R=۰/۷۳). نتیجه گیری تمرین مقاومتی با کاهش مایوستاتین و افزایش قدرت و حجم و سطح مقطع عضلانی همراه بود. این امر درنهایت ممکن است موجب کاهش آتروفی عضلانی وابسته به سن شود و در سلامت سالمندان مؤثر باشد.

Title: بررسی کیفی ارتباط با خداوند در دوره سالمندی

http://salmandj.uswr.ac.ir/article-1-1199-fa.html

اهداف تحقیق حاضر به منظور بررسی کیفی ارتباط با خدا در بین سالمندان انجام شد. رابطه انسان با خداوند به عنوان رابطه‌ای قدیمی، به رابطه انسان با دیگران و خود و هستی جهت می‌دهد. افرادی که با خدا ارتباط مثبتی داشته باشند در مقابل حوادث و مشکلاتی که با آن مواجه می‌شوند، اضطراب و آشفتگی روانی کمتری دارند.  مواد و روش ها در راستای رسیدن به هدف تحقیق و پاسخ‌گویی به سؤال‌های تحقیق، در فرایند روش تحقیق زمینه‌یابی(گراندد تئوری)، پرسش‌نامه باز پاسخ‌دهی طراحی و در قالب مصاحبه نیمه‌ساختاریافته اجرا شد. نمونه تحقیق نظری بود و تا رسیدن به اشباع نظری نمونه‌گیری ادامه یافت. درمجموع، نمونه تحقیق شامل 18 نفر از سالمندان در سنین 60 تا 81 سال بودند.  یافته ها نتایج به‌دست‌آمده شامل 105 مفهوم و 24 مقوله فرعی و 5 مقوله اصلی بود. مقوله‌های به‌دست‌آمده در متغیرهای شرایط علّی، پدیده، محتوا (زمینه)، شرایط میانجی، استراتژی کنش/ کنش متقابل و پیامد تحلیل شدند. درنهایت از رابطه بین مقوله‌ها الگویی ارائه شد.  نتیجه گیری الگوی نهایی ارائه‌شده حاکی از تنوع گسترده در کیفیت و کمیّت ارتباط با خدا بود. نقش عوامل عاطفی و شناختی و اجتماعی در بروز این رفت ار(ارتباط با خدا) شفاف است. با توجه به شرایط میانجی و پیامدهای ادراک‌شده از ارتباط با خداوند، چگونگی تضعیف یا تقویت ارتباط با خداوند ترسیم شد.

Title: مقایسه فراوانی عوامل خطر بروز یبوست در سالمندان با و بدون یبوست در بیمارستان حضرت رسول (ص)

http://salmandj.uswr.ac.ir/article-1-1201-fa.html

اهداف شناخت عوامل خطر و شیوع آن‌ها در بروز یبوست سالمندان در این مطالعه مدنظر بود. یبوست مشکل شایعی در سالمندان است و در سالمندان آسیب‌پذیر به عوارضی نظیر احتباس ادراری و بی‌اختیاری سرریز منجر می‌شود. توجه به گرفتن شرح حال دقیق برای شناسایی عوامل خطر، از مصرف بی‌رویه مسهل و عوارض آن در این گروه سنی جلوگیری خواهد کرد. مواد و روش ها این مطالعه مورد شاهدی بر روی صد بیمار 65 سال و بالاتر انجام شد. 50 نفر از آن‌ها یبوست داشتند و 50 نفر یبوست نداشتند که به کلینیک بیمارستان حضرت رسول اکرم مراجعه کرده بودند. برای تجزیه‌وتحلیل آماری از نرم‌افزار آماری 16.SPSS v استفاده شد. یافته ها نتایج حاصل از مطالعه حاضر نشان داد که فراوانی عواملی نظیر مصرف چندین دارو، مصرف داروهای بلاکرکانال کلسیم، مصرف داروهای آنتی‌کولینرژیک، تحرک نداشتن، دهیدراتاسیون، رژیم غذایی کم‌فیبر و نیز بیماری‌هایی همچون دیابت، هیپوتیروئیدیسم، سکته مغزی، بدخیمی، نارسایی کلیوی و پارکینسون به طور معنی‌داری در سالمندان دچار یبوست بیش از سایر سالمندان بدون این مشکل وجود دارد. از سوی دیگر، فراوانی مصرف مواد مخدر و افسردگی در دو گروه اختلاف قابل توجهی نداشت.  نتیجه گیری شناخت عوامل مؤثر در بروز یبوست در جمعیت سالمندان و سنجش فراوانی این عوامل اهمیت بسزایی در درمان و بهبود اجابت مزاج ایشان دارد و با حذف عوامل خطر می‌توان از مصرف بی‌رویه مسهل‌ها در این جمعیت آسیب‌پذیر جلوگیری کرد.

Title: پیش‌بینی رضایت از زندگی بر اساس ابعاد هوش معنوی و سرمایه روان‌شناختی در سالمندان

http://salmandj.uswr.ac.ir/article-1-1223-fa.html

اهداف سالمندی یکی از مراحل مهم زندگی است که با پیشرفت علم و بهبود شرایط بهداشتی، آمار سالمندان در جوامع گوناگون روبه‌افزایش است. از این رو برای ارتقای سازگاری سالمندان، توجه به رضایت از زندگی آنان لازم است. بنابراین پژوهش حاضر به منظور پیش‌بینی رضایت از زندگی بر اساس هوش معنوی و سرمایه روان‌شناختی در سالمندان انجام شد.  مواد و روش ها روش پژوهش توصیفی از نوع همبستگی بود. جامعه آماری ۱۸۰ سالمند ساکن در خانه‌های سالمندان شهرستان کاشان بودندکه به روش نمونه­‌گیری تصادفی ساده و با استفاده از جدول مورگان، ۱۲۳ نفر انتخاب شدند. داده‌هابا استفاده پرسش‌نامه های رضایت از زندگی، هـوش معنـوی و سـرمایه روان‌شـناختی جمع‌آوری و به کمک آزمون‌های ضریب همبستگی پیرسون و تحلیل رگرسیون چندگانه تحلیل شدند. یافته‌ها میانگین و انحراف معیار سن سالمندان (۵/۲±۷۰)، درک و ارتباط با هستی (۱۴/۷±۶۲/۱۸)، زندگی معنوی (۶/۷۵±۵۱/۱۱)، خودکارآمدی (۴/۱۴±۲۴/۱۴)، امیدواری (۱/۵۸±۲۲/۸۳)، تاب‌آوری (۴/۳۸±۲۱/۰۸)، خوش‌بینی (۵/۰۴±۲۸/۳۳) و رضایت زندگی (۲۱/۷۱±۲/۷۵) بود. نتایج پژوهش نشان داد که ابعاد هوش معنوی و سرمایه روان‌شناختی سطح رضایت از زندگی سالمندان را به طور معناداری پیش بینی می‌کنند (۰/۰۱>P) و ابعاد درک و ارتباط با هستی و امیدواری سهم بیشتری در پیش‌بینی رضایت از زندگی سالمندان دارند (۰/۰۱>P). نتیجه گیری نتایج پژوهش حاضر حاکی از این است که بخشی از تغییرات رضایت از زندگی سالمندان به کمک ابعاد هوش معنوی و سرمایه روان‌شناختی قابل پیش‌بینی است. بنابراین به منظور افزایش سطح رضایت از زندگی سالمندان پیشنهاد می‌شود که زمینه ارتقای سطح هوش معنوی و سرمایه روان‌شناختی آنان فراهم شود.

Title: مقایسه سطوح پلاسمایی پروتئین واکنش‌گر C، اینترلوکین-10 و اینترلوکین-15 در زنان یائسه فعال و غیرفعال

http://salmandj.uswr.ac.ir/article-1-1241-fa.html

اهداف سطوح بالای سایتوکاین‌های ضد التهابی اینترلوکین-10 و اینترلوکین-15 و سطح پایین پروتئین واکنش‌گر C با طول عمر بالاتر مرتبط است. تأثیر فعالیت ورزشی منظم روی این سایتوکاین‌ها در زنان یائسه به‌خوبی بررسی نشده است. مواد و روش ها 15 زن یائسه فعال و 15 زن یائسه غیرفعال با دامنه سنی 50 تا 60 سال به‌طور تصادفی برای پژوهش انتخاب شدند. معیارهای پژوهش برای گروه غیرفعال شامل عدم ابتلا به بیماری‌های مزمن، عدم استعمال دخانیات، عدم جراحی در یک سال اخیر، سپری‌شدن یک سال از زمان یائسگی و برای گروه فعال داشتن فعالیت ورزشی منظم با شدت متوسط (هفته‌ای سه جلسه در حداقل شش ماه و حداکثر یک سال گذشته) بود. فعالیت‌های ورزشی گروه فعال ترکیبی از پیاده‌روی، جاگینگ و تمرینات مقاومتی بود. پس از 12 ساعت ناشتابودن نمونه‌ها از آن‌ها نمونه خونی از سیاهرگ دست چپ در حالت نشسته و استراحت گرفته شد. سرم با دستگاه سانتریفیوژ در دور 4 هزار، به مدت 5 دقیقه از خون جدا شد و برای اندازه‌گیری اینترلوکین-10 و اینترلوکین-15 و پروتئین واکنش‌گر C استفاده شد. برای مقایسه نتایج در گروه فعال و غیرفعال از آزمون تی مستقل استفاده شد. تمام محاسبات آماری در سطح معناداری 05/0 انجام گرفت. یافته ها نتایج نشان داد فعالیت ورزشی منظم باعث کاهش معنادار پروتئین واکنش‌گر C، افزایش معنادار اینترلوکین-15 و افزایش غیرمعنادار اینترلوکین-10 در زنان یائسه فعال نسبت به زنان یائسه غیرفعال شده است. نتیجه گیری احتمالاً فعالیت ورزشی منظم از طریق کاهش التهاب و افزایش سایتوکاین‌های ضدالتهابی زنان یائسه مسن در برابر بیماری‌های مزمن مقابله می‌کند و شاید از این طریق در افزایش طول عمر مؤثر باشد.

Title: ارزیابی داروخانه‌های بیمارستانی شهر شیراز با معیار داروخانه دوستدار سالمند

http://salmandj.uswr.ac.ir/article-1-1272-fa.html

اهداف با توجه به تغییرات ساختار جمعیتی کشور، روزبه‌روز تعداد افراد مراجعه‌کننده به داروخانه‌ها در حال افزایش است. همچنین با توجه به افزایش روزافزون جمعیت سالمندان و متعاقب آن افزایش نیاز به دارو و نیز مراجعه بیشتر افراد سالمند به داروخانه ها، این مطالعه با هدف ارزیابی داروخانه‌های شهر شیراز با معیار داروخانه دوستدار سالمند انجام شد. مواد و روش ها این مطالعه به صورت مقطعی، توصیفی‌تحلیلی در سال ۱۳۹۵ انجام شده است. جامعه مورد مطالعه تمام ۳۳ داروخانه بیمارستانی شهر شیراز بود که به صورت سرشماری وارد مطالعه شدند. ابزار جمع‌آوری داده‌ها چک‌لیست محقق ساخته با هدف «قابلیت تبدیل داروخانه‌های شهر شیراز به داروخانه‌های دوستدار سالمند» بود که پایایی آن با استفاده از ضریب همیستگی ۰/۸۵ و روایی آن از طریق تأیید متخصصان برآورد شد. تجزیه‌و‌تحلیل داده‌ها با استفاده از نرم افزار SPSS نسخه ۲۳ برای انجام آزمون‌های کروسکال والیس، من ویتنی و کای دو استفاده شد. یافته ها متوسط نمره قابلیت تبدیل داروخانه‌ها به داروخانه‌های دوستدار سالمند ۵۰/۵۲ به دست آمد که بیانگر سطح متوسط داروخانه‌های شهر شیراز از لحاظ دوستدار سالمند بودند. نتایج حاصل از آزمون کروسکال والیس برای تعیین ارتباط نمره نهایی داروخانه‌های دوستدار سالمند با مالکیت داروخانه نیز نشان داد که ارتباط معناداری بین این دو متغیر وجود نداشت. همچنین ارتباط بین نوع داروخانه و نمره نهایی قابلیت تبدیل داروخانه‌های دوستدار سالمند نیز معنی‌دار نشد. نتیجه گیری با توجه به مسن شدن جامعه، همچنین سطح متوسط داروخانه‌های سطح شهر شیراز از لحاظ دوستدار سالمند بودن، آمادگی داروخانه‌های شهر شیراز برای ارائه خدمات به سالمندان یکی از چالش‌برانگیزترین موضوعات در بلندمدت است. با سیاست‌گذاری صحیح و مناسب بر سیستم‌های مراقبت بهداشتی و درمانی، به ویژه داروخانه‌ها می‌توان مشکلات سالمندان را در آینده کاهش داد.
